# Trends in Incidence and Clinical Outcomes of *Clostridioides difficile* Infection, Hong Kong

**DOI:** 10.3201/eid2712.203769

**Published:** 2021-12

**Authors:** Cosmos L.T. Guo, Thomas N.Y. Kwong, Joyce W.Y. Mak, Lin Zhang, Grace C.Y. Lui, Grace L.H. Wong, Margaret Ip, Jun Yu, Joseph J.Y. Sung, William K.K. Wu, Sunny H. Wong

**Affiliations:** The Chinese University of Hong Kong, Hong Kong, China (C.L.T. Guo, T.N.Y. Kwong, J.W.Y. Mak, L. Zhang, G.C.Y. Lui, G.L.H. Wong, M. Ip, J. Yu, J.J.Y. Sung, W.K.K. Wu, S.H. Wong);; Lee Kong Chian School of Medicine, Nanyang Technological University, Singapore (J.J.Y. Sung, S.H. Wong)

**Keywords:** *Clostridioides difficile*, pseudomembranous colitis, antimicrobial resistance, epidemiology, surveillance, Hong Kong, bacteria, *Suggested citation for this article*: Guo CLT, Kwong TNY, Mak JWY, Zhang L, Lui GCY, Wong GLH, et al. Trends in incidence and clinical outcomes of *Clostridioides difficile* infection, Hong Kong. Emerg Infect Dis. 2021 Dec [*date cited*]. https://doi.org/10.3201/eid2712.203769

## Abstract

We conducted a territorywide survey to investigate the epidemiology, risk factors, and clinical outcomes of *Clostridioides difficile* infection (CDI) among hospitalized patients in Hong Kong. A total of 17,105 cases of CDI were identified, of which 15,717 (91.9%) were healthcare-associated and 1,025 (6.0%) were community-associated. Although CDI incidence increased substantially from 2006 to 2017, it plateaued in 2018 and 2019. The 30-day mortality rates decreased from 20.1% in 2015 to 16.8% in 2019, whereas the 60-day recurrence rates remained constant at ≈11% during the study period. Cross-correlation statistic showed significant correlations between incidence trend and overall antimicrobial drug use (r = 0.865, p<0.001), which has decreased as a result of an antibiotic stewardship program initiated in 2017. Our data suggest a turning point in *C. difficile* epidemiology that could be related to the changing pattern of antimicrobial drug use.

## Abstract 2

Surveillance of *C. difficile* infections suggests correlation of incidence to antibiotic stewardship programs.

*Clostridioides difficile* infection (CDI) is a common nosocomial disease; symptoms range from mild diarrhea to life-threatening colitis and toxic megacolon. CDI is associated with a high mortality rate, particularly for patients >75 years of age (*1*). Epidemiologic studies have identified its substantial incidence especially in the United States and in many countries in Europe (*2*,*3*). Recent data suggested that its overall incidence in some of these countries have reached a plateau. For instance, the US Centers for Disease Control and Prevention (CDC) reported a decrease in CDI incidence from 2014 to 2017 (*2*), whereas the overall CDI incidence in Sweden has decreased by 22% from 2012 to 2016 (*4*). These declines are often attributed to the implementation of antibiotic stewardship programs. Nonetheless, community-acquired CDI (CA-CDI) represents a growing threat; incidence of CA-CDI doubled from 2011–2015 (*5*).

The epidemiologic patterns in different geographic regions are highly dynamic. Outbreaks of CDI in North America and Europe were once predominantly caused by the *C*. *difficile* ribotype 027 (*6*), which was rarely reported about in Asia (*7*). Instead, *C*. *difficile* ribotype 017 has been the predominant strain in Asia (*8*). Other toxigenic strains, such as *C*. *difficile* ribotype 369, which was associated with multiple epidemics in Japan, have been reported in various Southeast Asia countries (*9*), whereas *C. difficile* ribotype 002 was reported to be common in Hong Kong, China, and was associated with increased virulence (*10*,*11*). Continuous surveillance, therefore, is important to prevent outbreaks of CDI. However, epidemiologic data of CDI in Asia remain sparse. We have previously characterized the molecular and antimicrobial susceptibility patterns of prevalent *C*. *difficile* ribotypes in Hong Kong (*12*). We also conducted an observational study to investigate CDI disease burden and clinical outcomes among hospitalized patients in Hong Kong, which showed a rapidly increasing incidence until 2014 (*13*). In this study, we continued to update the epidemiologic pattern of CDI among hospitalized patients in Hong Kong and characterize CDI-associated risk factors and clinical outcomes.

We conducted this study in accordance with the Declaration of Helsinki (2013 version). The Joint Clinical Research Ethics Committee of the Chinese University of Hong Kong and Hospital Authority New Territory East Cluster approved the study. All clinical data were anonymized by the Clinical Data Analysis and Reporting System (CDARS), and all potential identifiers were removed upon return of database searches.

## Methods

### Study Population and Data Extraction

We identified digital records of all patients hospitalized in public hospitals with a laboratory-confirmed diagnosis of CDI in Hong Kong during January 1, 2015–December 31, 2019, from CDARS, a database of public hospital patient records managed by the Hong Kong Hospital Authority. We obtained clinical data including the patient demographics, laboratory results, drug prescription records, clinical outcomes, and diagnoses of underlying conditions. Patient demographic data include age and gender. We identified relevant diagnoses using codes from the International Classification of Diseases, 9th Revision, in accordance with the Charlson Comorbidity Index (*14*). We also obtained data on antimicrobial drug use and other drug use within 8 weeks before CDI diagnosis.

### Case Identification and Definitions

We defined a CDI case as positive result obtained from culture, toxin, or molecular assay for a diarrheal fecal specimen collected from inpatient residents >18 years of age. As described previously (*13*), patients with samples obtained >48 hours after admission or those who were hospitalized in a healthcare facility within the previous 4 weeks were classified as cases of healthcare-associated CDI (HA-CDI). We defined community-associated CDI (CA-CDI) as patients who had not been hospitalized in a healthcare facility within the previous 12 weeks. We defined patients who had been hospitalized in a healthcare facility within the previous 4–12 weeks as indeterminate. We classified patients with a maximum leukocyte count >15,000 cells/μL or >50% increase in serum creatinine level as having cases of severe CDI, as defined by the Infectious Diseases Society of America (*15*). We defined refractory disease as a nonresponding disease requiring >14 days of continued treatment and a treatment period as a period during which records of drug prescription records indicate continuous antimicrobial treatment with <3 days of interruption.

### Antimicrobial Drug Use Data

Because the use of antimicrobial drugs is a major risk factor for CDI, we extracted from the data the overall corporate use of antimicrobial drugs in public hospitals in Hong Kong and analyzed. The data were recorded as daily defined doses (DDDs), which is the assumed average maintenance dose per day for each drug. These can demonstrate the absolute changes in use, as well as DDDs per 1,000 bed-days occupied (DDD/1,000 BDO), which can demonstrate changes in use relative to hospital occupancy. Broad-spectrum antimicrobial drugs include cefepime, ceftazidime, cefotaxime, cefoperazone/sulbactam, piperacillin, piperacillin/tazobactam, carbapenems, and quinolones. We determined the risk for CDI for each drug class on the basis of its association with CDI (*1*). High-risk drugs were lincosamides, cephalosporins, fluoroquinolones, amoxicillin, and ampicillin. Medium-risk drugs included sulphonamides and macrolides. Low-risk drugs included tetracyclines (*1*).

### Statistical Analysis

We analyzed data with R version 3.6.0. (R Foundation for Statistical Computing, https://www.r-project.org) We defined annual crude incidence of CDI as the number of patients given a diagnosis of CDI per 100,000 adult population, using data obtained from the Hong Kong Census and Statistics Department. We analyzed potential predictors for 30-day mortality rate and 60-day recurrence rate using univariate and multivariate forward Wald logistic regression. We used Cox proportional hazard regression to identify factors that decreased the time to recurrence after an episode. We used χ^2^ test of proportion to compare differences in incidences, mortality rates, and recurrence rates. We used cross-correlation to identify correlation between CDI incidence and antimicrobial drug use and p = 0.05 as a measure of statistical significance.

## Results

### Disease Burden and Incidence

During 2015–2019, we identified 17,105 cases of CDI among hospitalized patients in Hong Kong (average 3,421 cases/year). Of these cases, 15,717 (91.9%) were HA-CDI and 1,025 (6.0%) were CA-CDI. The remaining 363 cases (2.1%) were indeterminate (Table 1; Appendix Figure 1).

**Table 1:** Crude incidence of *Clostridioides difficile* infections, by epidemiologic category, Hong Kong, China, 2015–2019*

Year	Adult population	No. cases				Incidence†		
		Overall	HA-CDI	CA-CDI		Overall	HA-CDI	CA-CDI
2015	6,247,460	3,160	2,921	181		50.6	46.8	2.9
2016	6,301,560	3,303	3,058	185		52.4	48.5	2.9
2017	6,357,420	3,618	3,303	231		56.9	52.0	3.6
2018	6,410,080	3,557	3,248	223		55.5	50.7	3.5
2019	6,481,000	3,467	3,187	205		53.5	49.2	3.2

*Adult is defined as a person >18 years of age. CA-CDI, community-associated *Clostridioides difficile* infection; HA-CDI, healthcare-associated *Clostridioides difficile* infection. †No. cases/100,000 adults.

Although a rapid increase of CDI incidence was observed during 2006–2014, the crude incidence of CDI in Hong Kong remained relatively stable and the average annual percentage change (APC) from 2015 to 2019 showed a modest increase of 1.53% (Table 1; Figure, panel A). Among the different age groups, the CDI incidence showed a significant decrease for patients >75 years of age (Figure, panel B). We observed a similar downward trend in the overall incidences of HA-CDI (Figure, panel A), the first time since the start of our previous study in 2006 (*13*). However, this decrease was not evident in the younger patient groups between 2015 and 2019, despite these groups only representing a minor proportion of CDI incidence (Table 1; Figure).

**Figure:**
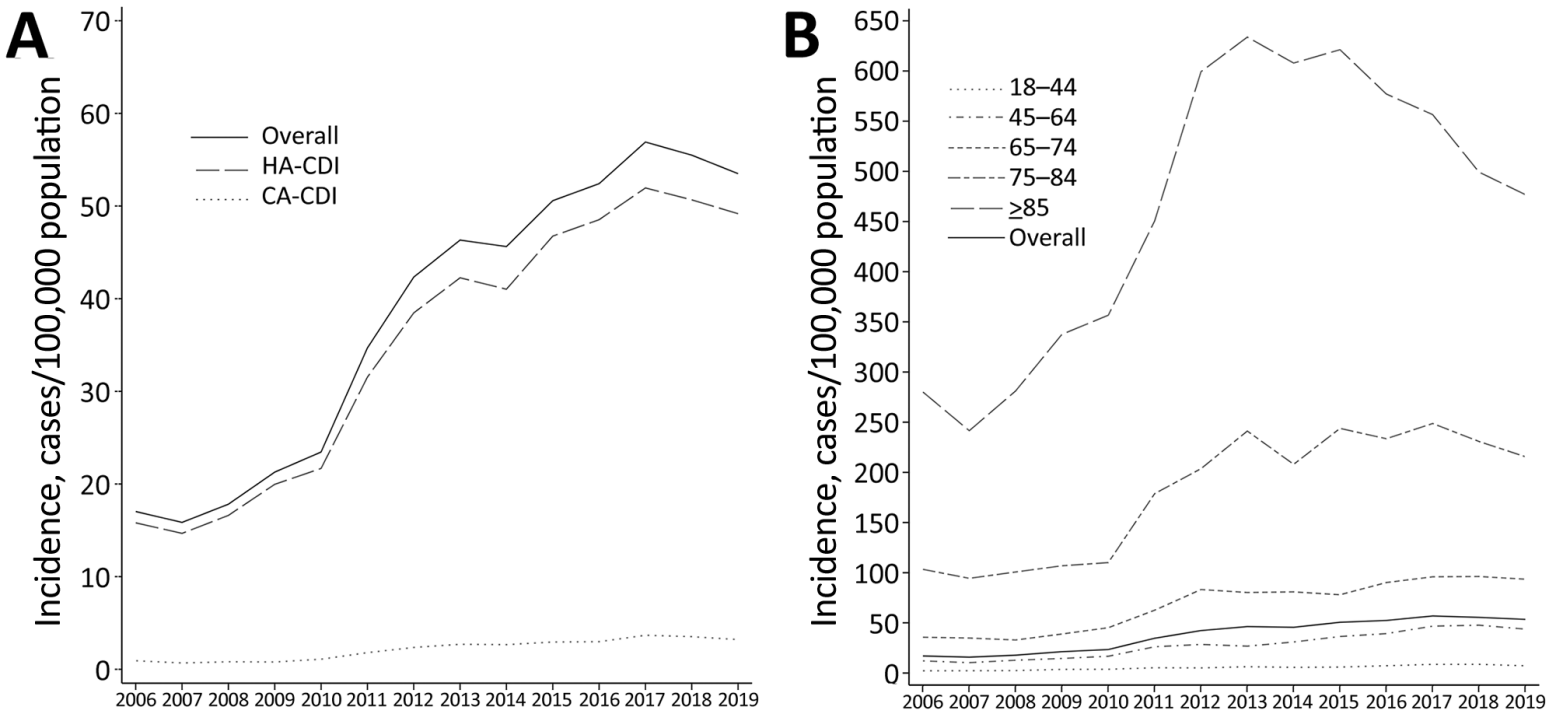
*Clostridioides difficile* infections in adults, Hong Kong, 2006–2019. Data for 2006–2014 were acquired from a previous study (*13*). A) Crude incidence of healthcare-associated and community-associated *C. difficile* infections. B) Incidence of infections by age group.

The median age of patients was 77 years (interquartile range [IQR] 63–86 years); 51.8% were female and 48.2% male (Table 2). The number of patients from old-age homes has significantly decreased, from 29.2% in 2015 to 22.8% in 2019 (p<0.001) (Table 3), compared with an average of 30.2% in the period 2006–2014. Most patients have taken high-risk antimicrobial drugs (81.4%), broad-spectrum antimicrobial drugs (59.3%), or proton-pump inhibitors (PPI) (62.1%) within 8 weeks before diagnosis of CDI (Table 2). Of note, during the period of 2015–2019, the proportion of severe CDI has decreased from 38.2% to 31.2% of patients (p<0.001) (Table 3).

**Table 2:** Characteristics, outcomes, and procedures of patients with *Clostridioides difficile* infections, Hong Kong, China, 2015–2019*

Characteristic	Overall	CA-CDI	HA-CDI	Indeterminate
All patients	17,105	1,025 (6.0)	15,717 (91.9)	363 (2.1)
Age, y				
Median (IQR)	77 (63–86)	74 (58–85)	77 (63–86)	78 (62–86)
<44	1,056 (6.2)	138 (13.5)	901 (5.7)	17 (4.7)
45–64	3,679 (21.5)	221 (21.6)	3,375 (21.5)	83 (22.9)
65–74	3,027 (17.7)	173 (16.9)	2,793 (17.8)	61 (16.8)
75–84	4,340 (25.4)	207 (20.2)	4,044 (25.7)	89 (24.5)
>85	5,003 (29.2)	286 (27.9)	4,604 (29.3)	113 (31.1)
Sex				
M	8,252 (48.2)	442 (43.1)	7,642 (48.6)	168 (46.3)
F	8,853 (51.8)	583 (56.9)	8,075 (51.4)	195 (53.7)
Admission from OAH	4,321 (25.3)	209 (20.4)	4,003 (25.5)	109 (30.0)
IDSA-defined severe disease	5,871 (35.8)	295 (29.0)	5,482 (36.5)	94 (26.2)
Diagnostic test				
Bacterial culture	8,191 (40.9)	468 (39.4)	7,560 (41.0)	163 (38.6)
Nucleic acid amplification test	8,994 (44.9)	547 (46.0)	8,261 (44.8)	186 (44.1)
Toxin detection	2,855 (14.2)	173 (14.6)	2,609 (14.2)	73 (17.3)
Antimicrobial drug use				
High-risk drugs	13,932 (81.4)	272 (26.5)	13,519 (86.0)	141 (38.8)
Medium-risk drugs	1,562 (9.1)	23 (2.2)	1,526 (9.7)	13 (3.6)
Low-risk drugs	4,286 (25.1)	17 (1.7)	4,257 (27.1)	12 (3.3)
Broad-spectrum drugs	10,147 (59.3)	94 (9.2)	9,986 (63.5)	67 (18.5)
Use of other drugs				
Proton pump inhibitor	10,614 (62.1)	201 (19.6)	10,255 (65.2)	158 (43.5)
H2 antagonist	4,950 (28.9)	158 (15.4)	4,722 (30.0)	70 (19.3)
Corticosteroid	4,477 (26.2)	81 (7.9)	4,350 (27.7)	46 (12.7)
Underlying conditions				
Myocardial infarction	1,212 (7.1)	37 (3.6)	1,144 (7.3)	31 (8.5)
Congestive heart failure	2,407 (14.1)	64 (6.2)	2,292 (14.6)	51 (14.0)
Peripheral vascular disease	556 (3.3)	10 (1.0)	536 (3.4)	10 (2.8)
Cerebrovascular disease	3,051 (17.8)	91 (8.9)	2,887 (18.4)	73 (20.1)
Chronic pulmonary disease	1,937 (11.3)	84 (8.2)	1,806 (11.5)	47 (12.9)
Mild liver disease	338 (2.0)	18 (1.8)	301 (1.9)	19 (5.2)
Severe liver disease	243 (1.4)	11 (1.1)	221 (1.4)	11 (3.0)
Diabetes mellitus	3,624 (21.2)	131 (12.8)	3,414 (21.7)	79 (21.8)
Diabetes mellitus with complications	1,492 (8.7)	44 (4.3)	1,406 (8.9)	42 (11.6)
Moderate/severe kidney disease	3,363 (19.7)	94 (9.2)	3,178 (20.2)	91 (25.1)
Nonmetastatic cancer	3,403 (19.9)	75 (7.3)	3,268 (20.8)	60 (16.5)
Metastatic cancer	970 (5.7)	21 (2.0)	932 (5.9)	17 (4.7)
HIV	16 (0.1)	1 (0.1)	15 (0.1)	0
Paraplegia	356 (2.1)	12 (1.2)	336 (2.1)	8 (2.2)
Connective tissue disease	174 (1.0)	15 (1.5)	152 (1.0)	7 (1.9)
Dementia	863 (5.0)	47 (4.6)	793 (5.0)	23 (6.3)
Peptic ulcer	867 (5.1)	24 (2.3)	826 (5.3)	17 (4.7)
Outcomes				
Episode death	3,220 (18.8)	73 (7.1)	3,117 (19.8)	30 (8.3)
30-day mortality	3,225 (18.9)	87 (8.5)	3,100 (19.7)	38 (10.5)
60-day mortality	4,738 (27.7)	117 (11.4)	4,562 (29.0)	59 (16.3)
30-day recurrence	1,968 (11.5)	0	1,947 (12.4)	21 (5.8)
Refractory disease	2,155 (12.6)	59 (5.8)	2,064 (13.1)	32 (8.8)
Procedures				
Partial colectomy	3 (0.0)	1 (0.1)	2 (0.0)	0
Left colectomy	3 (0.0)	0	3 (0.0)	0
Right colectomy	6 (0.0)	0	6 (0.0)	0
Sigmoid colectomy	1 (0.0)	0	1 (0.0)	0
Total colectomy	4 (0.0)	1 (0.1)	3 (0.0)	0
Fecal microbiota transplant	3 (0.0)	0	3 (0.0)	0

*Values are no. (%) patients except as indicated. CA-CDI, community-associated* Clostridioides difficile *infection; HA-CDI, healthcare-associated* Clostridioides difficile *infection; IDSA, Infectious Diseases Society of America; IQR, interquartile range; OAH, old age home.

**Table 3:** Logistic regression analysis of potential independent variables associated with 30-day mortality for *Clostridioides difficile* infection, Hong Kong, China, 2015–2019*

Variable	Univariate			Multivariate	
	OR (95% CI)	p value		OR (95% CI)	p value
Age, y					
<44	Referent	NA		Referent	NA
45–64	3.115 (2.301–4.322)	<0.001		2.458 (1.794–3.449)	<0.001
65–74	4.203 (3.105–5.829)	<0.001		3.203 (2.334–4.502)	<0.001
75–84	6.237 (4.643–8.595)	<0.001		5.384 (3.944–7.531)	<0.001
>85	7.986 (5.959–10.986)	<0.001		7.633 (5.583–10.70)	<0.001
Male sex	1.124 (1.041–1.213)	0.0029		1.221 (1.121–1.330)	<0.001
IDSA-defined severe disease	2.296 (2.121–2.486)	<0.001		2.159 (1.986–2.347)	<0.001
Healthcare-associated disease	2.483 (2.066–3.010)	<0.001		1.378 (1.119–1.708)	0.003
Admission from OAH	1.716 (1.579–1.864)	<0.001		1.327 (1.203–1.463)	<0.001
Diagnostic test					
Bacterial culture	Referent	NA		Referent	NA
Nucleic acid amplification test	1.070 (0.991–1.157)	0.0845		1.046 (0.962–1.138)	0.294
Toxin detection	0.995 (0.669–1.438)	0.9799		0.904 (0.592–1.343)	0.629
Antimicrobial drug use					
High-risk drugs	2.243 (1.990–2.534)	<0.001		0.753 (0.603–0.939)	0.012
Medium-risk drugs	1.021 (0.893–1.164)	0.7602		1.988 (0.653–8.670)	0.281
Low-risk drugs	1.193 (1.094–1.300)	0.0001		0.841 (0.647–1.092)	0.193
Broad-spectrum drugs	1.673 (1.542–1.817)	<0.001		1.402 (1.244–1.581)	<0.001
Aminoglycosides	0.907 (0.773–1.059)	0.2223		1.026 (0.825–1.272)	0.815
Beta-lactamase inhibitor	2.276 (2.063–2.516)	<0.001		1.465 (1.047–2.052)	0.026
Carbapenem	1.229 (1.118–1.349)	<0.001		1.233 (0.967–1.571)	0.091
Cephalosporin	1.180 (1.087–1.279)	0.0001		0.936 (0.848–1.033)	0.187
Lincosamides	0.901 (0.554–1.400)	0.6580		1.126 (0.674–1.800)	0.634
Macrolides	1.411 (1.201–1.650)	<0.001		0.604 (0.140–1.812)	0.425
Penicillin	2.208 (2.003–2.438)	<0.001		1.165 (0.840–1.624)	0.362
Quinolones	1.115 (1.020–1.218)	0.0161		0.939 (0.843–1.045)	0.251
Sulphonamides	0.588 (0.465–0.734)	<0.001		0.395 (0.092–1.167)	0.138
Tetracyclines	1.291 (1.081–1.534)	0.0043		1.219 (0.955–1.548)	0.108
Use of other drugs					
Proton pump inhibitor	1.614 (1.486–1.755)	<0.001		1.182 (1.073–1.304)	<0.001
H2 antagonist	1.237 (1.136–1.346)	<0.001		1.225 (1.109–1.353)	<0.001
Corticosteroid	0.954 (0.876–1.038)	0.2759		0.919 (0.835–1.010)	0.080
Underlying conditions					
Myocardial infarction	1.268 (1.100–1.457)	0.0009		1.022 (0.871–1.195)	0.791
Congestive heart failure	1.632 (1.475–1.804)	<0.001		1.358 (1.208–1.525)	<0.001
Peripheral vascular disease	1.218 (0.989–1.489)	0.0588		0.980 (0.781–1.221)	0.859
Cerebrovascular disease	1.204 (1.092–1.325)	0.0002		1.021 (0.911–1.143)	0.722
Nonmetastatic cancer	1.498 (1.368–1.638)	<0.001		1.657 (1.468–1.869)	<0.001
Metastatic cancer	2.827 (2.466–3.237)	<0.001		2.627 (2.209–3.124)	<0.001
Diabetes mellitus	1.267 (1.157–1.386)	<0.001		1.052 (0.948–1.168)	0.339
Diabetes mellitus with complications	1.226 (1.076–1.394)	0.0020		1.205 (1.029–1.409)	0.020
Mild liver disease	1.005 (0.758–1.314)	0.9694		1.192 (0.829–1.690)	0.333
Severe liver disease	1.204 (0.878–1.623)	0.2360		1.643 (1.090–2.451)	0.016
Peptic ulcer	1.283 (1.087–1.508)	0.0029		1.060 (0.885–1.264)	0.523
Chronic pulmonary disease	1.302 (1.161–1.459)	<0.001		1.006 (0.883–1.144)	0.926
Moderate/severe kidney disease	1.203 (1.095–1.320)	0.0001		1.383 (1.231–1.553)	<0.001
Connective tissue disease	0.556 (0.338–0.865)	0.0139		0.970 (0.573–1.563)	0.904
Paraplegia	1.152 (0.885–1.482)	0.2810		1.054 (0.779–1.410)	0.726
Dementia	1.364 (1.158–1.600)	0.0002		1.033 (0.864–1.231)	0.718
HIV	0.287 (0.016–1.415)	0.2253		0.562 (0.030–3.080)	0.590

*IDSA, Infectious Diseases Society of America; NA, not applicable; OAH, old age home.

### Clinical Outcomes and Risk Factors

The 30-day all-cause mortality rates have decreased from 20.1% in 2015 to 16.8% in 2019 (p = 0.002), substantially lower than the previous decrease of 22.5% during the period 2006–2014 (Appendix Table 1) (*13*). Multivariate logistic regression analysis indicated that the main predictors for death in 30 days were advanced age (>85 years, adjusted OR [aOR] 7.23, 95% CI 5.29–10.12; 75–84 years, aOR 4.30, 95% CI 3.16–5.98) and metastatic tumor (aOR 2.63, 95% CI 2.21–3.12) (Table 3).

The 60-day recurrence rate remained at ≈11% (Appendix Table 1). Cox regression analysis showed that the main predictors of 60-day recurrence were healthcare-associated CDI (adjusted hazard ratio [aHR] 8.15, 95% CI 5.25–12.63) and use of quinolones (aHR 1.59, 95% CI 1.41–1.78) or broad-spectrum antibiotics (aHR 1.37, 95% CI 1.18–1.59) within 8 weeks before diagnosis of CDI (Appendix Table 2). Refractory disease rates decreased from 13.6% in 2015 to 11.3% in 2019, but the change was not statistically significant (p<0.076).

The number of patients who used tetracyclines within 8 weeks before CDI increased from 2.7% in 2015 to 6.2% in 2019 (p<0.001). In contrast, patient exposure to certain known risk factors has decreased during the period, including the use of H2 antagonists (from 35.2% to 25.0%; p<0.001), high-risk antibiotics (from 85.5% to 77.7%; p<0.001), penicillin group of drugs (from 74.2% to 68.5%; p<0.001) and fluoroquinolones (from 27.4% to 20.7%; p<0.001). The risk factor of having cerebrovascular accident as an underlying condition also decreased in these patients (from 21.9% to 15.9%; p<0.001) (Appendix Table 3).

### Comparison of HA-CDI and CA-CDI

The proportion of HA-CDI among all patients has remained at ≈90% and of CA-CDI at ≈5% (Appendix Figure). The year-to-year changes for the CA-CDI and HA-CDI incidence rates were not statistically significant, suggesting a static trend during the period (Table 1). Comparisons between the patients showed that HA-CDI patients had a median age of 77 (IQR 63–86) years, versus a median age of 74 (IQR 58–85) years for CA-CDI patients. Significantly more HA-CDI patients had severe CDI (36.5% vs 29.0%; p<0.001) and underlying conditions compared with CA-CDI patients (Table 2). HA-CDI patients were more likely to have exposure to high-risk antimicrobial drugs (86.0% vs. 26.5%; p<0.001) and broad-spectrum antimicrobials (63.5% vs 9.2%; p<0.001) within 8 weeks before CDI. The 30-day mortality rate was 19.7% for HA-CDI but 8.5% for CA-CDI patients (p<0.001), although both rates have decreased compared with the earlier period of 2006–2014 (*13*). Although the 30-day mortality rate for HA-CDI decreased from 2015 to 2019, we did not observe an obvious trend in mortality rate for CA-CDI patients (p = 0.354) (Appendix Table 1). The 60-day recurrence rate was 12.4% for HA-CDI, whereas none of the CA-CDI patients had a recurrence (Table 2).

### Data on Antimicrobial Drug Use

The overall use of antimicrobial drugs per year, measured as DDD/1,000 BDO, increased from 1,206 in 2006 to 4,747 in 2018 but then decreased to 3,968 in 2019 (Appendix Table 4). Annual use shared a significant correlation (r = 0.865; p<0.001) with the CDI incidence. In terms of DDD/1,000 BDO, we observed the highest levels of correlation for lincosamides (r = 0.907; p<0.001), carbapenems (r = 0.893; p<0.001) and sulphonamides (r = 0.872; p<0.001), followed by penicillin (r = 0.847; p<0.001) and quinolones (r = 0.825; p<0.001). Similar to the trend in overall antimicrobial drug use, all of these drugs had a decrease in use in 2019, after consistent increases from 2006–2018. We grouped ampicillin and amoxicillin together with other penicillin group drugs because their combined use attributed to 80% of all penicillin use from 2006–2019. In the same period, we observed increased use of tetracyclines, from 9.22 DDD/1,000 BDO in 2006 to 193.44 DDD/1,000 BDO in 2019.

## Discussion

In this study, we investigated the latest disease burden of CDI in Hong Kong to provide a complete picture of continual disease surveillance since 2006. Because the public hospitals provide >90% of inpatient medical service in Hong Kong, this study provides a comprehensive and near-complete data on the disease epidemiology among hospitalized patients in the territory. Our main finding was a decrease in the incidence of CDI in 2018 and 2019, in contrast with the distinctive increasing trend in 2006–2017 (*13*). The average APC during 2015–2019 showed a 1.53% increase, in contrast with the 13.76% increase for the average APC from 2006–2014. Year-to-year changes of crude CDI incidence in 2015–2019, except for 2016–2017, were statistically insignificant, suggesting that the incidence might have reached a plateau. Our reported incidence in Hong Kong (56.9 cases/100,000 population in 2017) was higher than incidence in the United Kingdom (24 cases/100,000 population in 2017), where CDI incidence has seen a decrease that was mostly attributed to a successful antimicrobial drug stewardship program (*16*,*17*). In comparison, Guh et al. reported an estimated crude incidence of 143.6 cases/100,000 population in the United States for 2017 (*2*). Despite the decrease in CDI incidence since 2011, it is still more than double the incidence in Hong Kong (*18*). In contrast, Liao et al. reported an overall crude CDI incidence in China during 2009–2016 was 34 cases/100,000 population, a relatively low value compared with our observations (*19*).

The trends in CDI incidence from 2006–2019 may partially be explained by changes in antimicrobial drug use. Usage rates of many drugs, such as penicillin, lincosamides (including clindamycin), quinolones, sulphonamides, and carbapenems have demonstrated significant correlations with CDI incidence. Penicillin, lincosamides, and quinolones are known to be high-risk for CDI, whereas sulphonamides and carbapenems carry a medium to low risk (*1*). The changes in penicillin use are likely the most relevant, because they were the most prescribed class of drugs during this period. We saw in the same period increased use of tetracyclines, which have been repeatedly demonstrated to have a relatively low risk for CDI (*20*). The changes in antimicrobial drug use may be attributable to Hong Kong’s antibiotic stewardship program, which was updated in 2017 (*21*). These changes are consistent with our observation on the decreased use of antimicrobial drugs, including the penicillin group, fluoroquinolones, and other high-risk drugs, in CDI patients from 2015–2019. By extrapolating the CDI incidence from 2006–2017 to predict incidence in 2018–2019, we observed that the predicted incidence in 2019 would be 20% higher than the actual incidence, showing that the potential effect antibiotic stewardship had on the status quo.

In addition to a decrease in incidence, we observed a decrease in 30-day mortality rates, for which there are myriad plausible explanations. The decrease may be attributed to improved effectiveness of treatment and management efforts for CDI, successful antibiotic stewardship programs, or increased use of fecal microbiota transplant as a treatment (*22*). Alternatively, a decrease in deaths may be attributable to a change in prevalence of ribotypes or their virulence, such as ribotype 002, which is common in Hong Kong. This difference may also explain the decrease in the relative proportion of severe CDI. Furthermore, ribotypes 002 and 017 are both virulent strains with high antimicrobial resistance (*8*,*10*,*12*), which may have been positively selected in the past because of excessive antimicrobial drug use. Now that the use of antimicrobial drugs has been declining, these strains may have seen a decline in prevalence, which lowered mortality rates. However, more data, such as those gathered through molecular typing and antibiotic resistance analysis, are required to validate this hypothesis.

Despite changes in CDI incidence, the proportion of CA-CDI cases of all CDI cases annually has remained steady from 2006–2019, at ≈4–5%. Risk factors for CA-CDI are unclear, although our data suggest that gastric acid suppression, antimicrobial drug use, and old age may be potential factors. Nonetheless, CA-CDI patients tend to be relatively younger. Previous studies have indicated that there is an increase in CA-CDI incidence and severe outcomes (*5*,*23*). Our study, however, indicates the proportion of CA-CDI has remained relatively constant and that their clinical outcomes are generally more favorable, including lower rates of mortality and recurrence, than outcomes for HA-CDI patients. Furthermore, we did not observe any significant trend of increase in CA-CDI cases. One potential explanation is that Hong Kong still has lower rates of inflammatory bowel disease compared with Western countries, despite an increasing trend in these illnesses over the past few decades (*22*,*24*).

The main strength of this study is that it is territorywide, driven by data extracted from all public hospitals in Hong Kong. All data, including demographics, laboratory results, drug prescription data, and procedures, were extracted from the public hospital database, thus reducing the possibility of recall bias. Furthermore, this study followed on our previous study (*13*) that investigated the epidemiology of CDI in Hong Kong during 2006–2014, providing a comprehensive epidemiologic pattern and comparisons for CDI in Hong Kong. Nonetheless, we acknowledge several limitations in our study. First, the data in this study were based only on inpatient data; we may have missed diagnoses made in outpatient clinics. Patients with mild symptoms could be sufficiently treated in outpatient clinics, which may result in an underestimation of the actual CDI incidence. Second, patient exposure to antimicrobial drugs and other drugs within 8 weeks of CDI was indicated as a logical indicator (i.e., true or false), regardless of dose, frequency, and prescription time, which may overestimate or underestimate the extent of the exposure. Third, there was a lack of data regarding changes in CDI diagnostic tests used from before 2015, making comparisons of CDI incidence unable to account for any shifts toward the use of the nucleic acid amplification test (NAAT), which is known to be a more sensitive test for CDI (*2*). Fortunately, the use of NAAT from 2015–2019 has remained within 40%–50%, which may mean a smaller impact on the trend of CDI incidence. Last, this is a retrospective study and there are unforeseeable covariates that were not adjustable or measurable, which may affect the analyses and results. Nevertheless, the epidemiology of CDI is dynamic, and changes can occur rapidly. We recommend continued surveillance of this infection in healthcare settings.

Appendix Additional information about trends in the disease prevalence and clinical outcomes of *Clostridioides difficile* infection, Hong Kong.
