# Supplementary material for: Trends in Incidence and Clinical Outcomes of Clostridioides difficile Infection, Hong Kong
Source: Emerg Infect Dis. 2021 Dec;27(12):3036–44. doi: 10.3201/eid2712.203769 (PMC8632188; doi:10.3201/eid2712.203769)
Supplement: Appendix — Additional information about trends in the disease prevalence and clinical outcomes of Clostridioides difficile infection, Hong Kong. [file 20-3769-Techapp-s1.pdf]

Article DOI: https://doi.org/10.3201/eid2712.213769

Trends in Incidence and Clinical Outcomes of *Clostridioides difficile* Infection, Hong Kong

Appendix

Appendix Table 1. Clinical outcomes of patients with *Clostridioides difficile* infections by epidemiological class, 2015-2019*

	Overall,%			HA-CDI, %			CA-CDI, %		
Year	30-day mortality	60-day recurrence	Refractory disease	30-day mortality	60-day recurrence	Refractory disease	30-day mortality	60-day recurrence	Refractory disease
2015	20.1	11.2	13.6	21.1	12.1	14.2	6.6	0.0	6.1
2016	19.9	12.6	12.5	20.8	13.4	12.8	6.5	0.0	6.5
2017	19.4	11.3	12.9	20.3	12.2	13.4	10.8	0.0	6.1
2018	18.2	11.2	12.8	19.2	12.1	13.4	7.6	0.0	4.9
2019	16.8	11.2	11.3	17.4	12.2	11.9	10.2	0.0	5.4
p value	0.002	0.281	0.076	0.002	0.452	0.079	0.354	NA	0.964

*CA-CDI, community-associated *C. difficile* infection; HA-CDI, healthcare-associated *C. difficile* infection.

Appendix Table 2. Cox proportional hazard regression analysis of potential independent variables associated with 60-day recurrence of *Clostridioides difficile* infections, Hong Kong, China, 2015-2019*

	Univariate		Multivariate	
Variable	HR (95% CI)	p value	HR (95% CI)	p value
Age				
<44	Reference	NA	Reference	NA
45–64	0.939 (0.771–1.144)	0.5316	0.894 (0.726–1.101)	0.290
65–74	0.944 (0.771–1.155)	0.5744	0.915 (0.735–1.14)	0.429
75–84	0.965 (0.796–1.171)	0.7185	0.98 (0.789–1.216)	0.854
≥85	0.916 (0.756–1.109)	0.3668	0.946 (0.757–1.182)	0.624
Male	1.123 (1.028–1.227)	0.0102	1.055 (0.960–1.160)	0.267
IDSA-defined severe disease	0.871 (0.791–0.960)	0.0053	0.827 (0.748–0.916)	<0.001
Healthcare-associated disease	8.767 (5.703–13.48)	0.0000	8.146 (5.253–12.63)	<0.001
Admission from OAH	1.083 (0.980–1.197)	0.1157	1.091 (0.971–1.226)	0.143
Diagnostic test				
Bacterial culture	Reference	NA	Reference	NA
Nucleic acid amplification test	0.906 (0.829–0.991)	0.0302	0.914 (0.832–1.005)	0.064
Toxin detection	0.801 (0.502–1.277)	0.3506	0.649 (0.382–1.102)	0.109
Antimicrobial drug use				
High-risk drugs	1.050 (0.936–1.178)	0.4075	0.519 (0.422–0.638)	<0.001
Medium-risk drugs	1.219 (1.057–1.406)	0.0064	1.427 (0.441–4.614)	0.553
Low-risk drugs	1.544 (1.406–1.696)	<0.001	1.223 (0.912–1.640)	0.178
Broad-spectrum drugs	1.687 (1.531–1.858)	<0.001	1.368 (1.178–1.590)	<0.001
Aminoglycosides	1.188 (1.008–1.401)	0.0401	0.776 (0.621–0.970)	0.026
Beta lactamase inhibitor	0.931 (0.846–1.025)	0.1445	1.220 (0.893–1.669)	0.212
Carbapenem	1.731 (1.569–1.909)	<0.001	1.204 (0.914–1.588)	0.187
Cephalosporin	1.169 (1.065–1.284)	0.0010	1.051 (0.942–1.173)	0.376
Lincosamides	1.109 (0.678–1.814)	0.6799	0.921 (0.561–1.513)	0.747
Macrolides	0.824 (0.663–1.024)	0.0806	0.572 (0.181–1.810)	0.342
Penicillin	0.895 (0.814–0.985)	0.0230	0.752 (0.554–1.020)	0.067
Quinolones	1.754 (1.598–1.924)	<0.001	1.585 (1.414–1.777)	<0.001
Sulphonamides	1.693 (1.417–2.022)	<0.001	0.891 (0.279–2.843)	0.846
Tetracyclines	0.693 (0.536–0.896)	0.0051	0.570 (0.422–0.771)	<0.001
Use of other drugs				
Proton pump inhibitor	1.417 (1.288–1.560)	<0.001	1.266 (1.135–1.413)	<0.001
H2 antagonist	1.094 (0.994–1.204)	0.0653	1.176 (1.061–1.303)	0.002
Corticosteroid	1.023 (0.926–1.131)	0.6527	0.888 (0.793–0.995)	0.041
Underlying conditions				
Myocardial infarction	1.341 (1.150–1.563)	0.0002	1.237 (1.045–1.465)	0.014

Variable	Univariate		Multivariate	
	HR (95% CI)	p value	HR (95% CI)	p value
Congestive heart failure	0.914 (0.802–1.042)	0.1789	0.831 (0.719–0.961)	0.013
Peripheral vascular disease	1.166 (0.925–1.471)	0.1944	0.938 (0.727–1.209)	0.620
Cerebrovascular disease	1.251 (1.122–1.393)	0.0001	1.140 (1.004–1.293)	0.043
Nonmetastatic cancer	1.080 (0.969–1.203)	0.1649	1.107 (0.970–1.265)	0.133
Metastatic cancer	0.629 (0.498–0.795)	0.0001	0.603 (0.462–0.789)	<0.001
Diabetes mellitus	1.038 (0.933–1.155)	0.4904	0.901 (0.798–1.018)	0.094
Diabetes mellitus with complications	1.123 (0.967–1.303)	0.1289	1.013 (0.850–1.206)	0.889
Mild liver disease	0.676 (0.462–0.988)	0.0433	0.813 (0.520–1.271)	0.364
Severe liver disease	0.667 (0.424–1.048)	0.0789	0.748 (0.434–1.291)	0.298
Peptic ulcer	1.158 (0.959–1.400)	0.1274	1.112 (0.912–1.356)	0.294
Chronic pulmonary disease	1.010 (0.879–1.160)	0.8893	1.121 (0.964–1.305)	0.138
Moderate/severe kidney disease	1.291 (1.164–1.431)	<0.001	1.289 (1.136–1.463)	<0.001
Connective tissue disease	0.674 (0.398–1.140)	0.1414	0.656 (0.377–1.141)	0.136
Paraplegia	1.285 (0.973–1.697)	0.0773	1.145 (0.838–1.564)	0.395
Dementia	1.057 (0.868–1.286)	0.5814	1.067 (0.867–1.313)	0.541
HIV	2.378 (0.892–6.343)	0.0835	2.199 (0.699–6.922)	0.178

*HR, hazard regression; NA, not applicable; OAH, old age home.

Appendix Table 3. Temporal change in prevalence of characteristics and exposure among patients with *Clostridioides difficile* infections, 2015-2019*

Percentage	2015	2016	2017	2018	2019	p value
Age						
<44	5.3	6.2	6.7	6.8	5.8	0.064
45-64	19.6	20.3	22.2	23.1	22.0	0.002
65-74	14.2	16.8	17.6	19.1	20.4	<0.001
75-84	29.0	26.4	25.4	23.7	22.8	<0.001
≥85	31.9	30.3	28.2	27.3	29.0	<0.001
Male sex	47.3	49.3	49.9	46.4	48.3	0.020
Severe disease	38.2	37.3	35.8	36.5	31.2	<0.001
Healthcare-associated disease	92.4	92.6	91.3	91.3	91.9	0.152
Admission from OAH	29.2	28.1	23.5	23.4	22.8	<0.001
Diagnostic test						
Bacterial culture	40.0	41.6	42.9	40.6	39.2	0.028
Nucleic acid amplification test	40.7	46.1	43.8	46.9	46.4	<0.001
Toxin detection	19.3	12.2	13.3	12.5	14.4	<0.001
Antimicrobial drug use						
High-risk drugs	85.5	82.5	80.0	82.0	77.7	<0.001
Medium-risk drugs	9.8	8.9	9.6	9.1	8.3	0.249
Low-risk drugs	23.7	22.6	23.9	27.2	27.7	<0.001
Broad-spectrum drugs	61.3	58.7	57.3	61.3	58.2	<0.001
Aminoglycosides	6.7	6.6	5.9	7.7	6.6	0.059
Beta-lactamase inhibitor	74.1	73.1	69.9	71.7	68.5	<0.001
Carbapenem	18.7	17.1	18.2	20.2	21.5	<0.001
Cephalosporin	35.0	32.8	28.1	30.7	28.4	<0.001
Lincosamides	0.8	0.8	0.4	0.7	1.0	0.071
Macrolides	5.8	5.4	5.9	4.8	4.0	0.001
Penicillin	74.2	72.9	69.8	71.3	68.5	<0.001

Quinolones	27.4	23.6	23.2	23.5	20.7	<0.001
Sulphonamides	4.3	3.8	3.8	4.4	4.5	0.473
Tetracyclines	2.7	2.9	4.2	5.6	6.2	<0.001
Use of other drugs						
Proton pump inhibitor	59.9	61.2	61.6	64.5	62.8	0.002
H2 antagonist	35.2	31.7	26.3	27.4	25	<0.001
Corticosteroid	26.8	21.6	26.9	28.1	27.2	<0.001
Comorbidities						
Myocardial infarction	7.7	7.3	6.6	7.2	6.7	0.316
Congestive heart failure	14.7	14.1	12.9	14.1	14.7	0.213
Peripheral vascular disease	3.6	3.4	3.5	2.7	3.1	0.192
Cerebrovascular disease	21.9	19.4	17.2	15.2	15.9	<0.001
Chronic pulmonary disease	11.7	12.4	12.3	10.1	10.1	<0.001
Mild liver disease	2.4	1.5	2.2	1.9	1.8	0.122
Severe liver disease	1.8	1.3	1.4	1.5	1.2	0.350
Diabetes mellitus	22.4	22.0	21.0	20.6	20.1	0.114
Diabetes mellitus with complications	9.1	10.0	8.7	8.4	7.5	<0.001
Moderate/severe kidney disease	17.7	20.2	20.1	20.2	19.9	0.046
Nonmetastatic cancer	19.9	19.1	20.2	20.3	19.9	0.736
Metastatic cancer	5.2	5.4	6.2	5.4	6.0	0.285
HIV	0.0	0.1	0.1	0.2	0.0	0.036
Paraplegia	3.0	1.8	2.0	1.7	2.0	<0.001
Connective tissue disease	1.3	1.1	1.0	0.7	1.0	0.195
Dementia	7.1	6.1	4.9	4.0	3.4	<0.001
Peptic ulcer	5.1	4.4	4.9	5.4	5.5	0.250

*p value was derived using χ^2 test. OAH, old age home.

Appendix Table 4. Antimicrobial drug use in public hospitals grouped by drug class, Hong Kong, 2006–2019*

	Daily defined dose/1,000 bed-days occupied													
Antimicrobial drug	2006	2007	2008	2009	2010	2011	2012	2013	2014	2015	2016	2017	2018	2019
Aminoglycoside	22.1	33.1	31.8	29.1	29.2	25.6	24.6	21.5	21.2	33.2	32.7	32.2	31.5	23.1
B-lactamase inhibitors	284.7	502.0	549.7	597.6	669.7	652.2	763.6	811.2	868.7	1,563.7	1,637.7	1,638.1	1,684.6	1,407.1
Carbapenem	13.0	26.4	26.6	34.2	36.8	40.7	49.2	56.6	63.6	112.6	124.1	131.6	154.7	129.7
Cephalosporin	149.9	218.8	221.6	215.3	220.7	218.3	213.3	201.1	194.8	283.9	274.1	244.3	240.2	201.0
Daptomycin	0.0	0.0	0.1	0.2	0.4	0.4	0.8	1.3	1.2	2.6	2.8	3.8	3.8	3.4
DHFRi	10.2	15.2	14.9	15.3	14.8	15.9	15.5	18.5	19.0	27.3	26.6	27.6	30.5	28.5
Phosphomycin	0.0	0.0	0.0	0.0	0.0	0.0	0.0	0.1	0.1	0.1	0.2	0.2	0.5	0.6
Glycopeptide	8.7	14.6	14.2	15.5	17.2	20.0	21.1	24.2	26.1	45.8	50.6	52.3	58.4	49.5
Lincosamide	1.3	2.6	3.6	4.0	5.1	5.7	4.8	5.8	5.8	8.4	9.5	8.8	10.1	7.9
Macrolide	56.9	90.2	87.9	97.4	117.1	107.5	114.7	107.1	92.5	132.4	134.0	134.4	111.3	83.9
Monobactam	0.0	0.0	0.0	0.0	0.0	0.0	0.0	0.0	0.0	0.0	0.1	0.1	0.1	0.1
Nitrofuran	8.8	14.0	14.6	13.2	11.3	11.3	10.7	9.7	9.6	16.1	16.8	14.9	16.0	13.7
Oxazolidinone	0.6	0.7	0.9	1.5	1.3	1.5	2.1	2.5	3.3	5.3	5.2	5.5	5.7	6.1
Penicillin	499.8	815.0	851.8	869.0	921.8	884.8	962.3	980.7	1,019.7	1,764.2	1,814.9	1,799.3	1,826.6	1,515.8
Polymixin	0.1	0.0	0.2	0.7	1.0	1.3	1.8	3.2	5.7	7.9	6.6	5.9	5.1	5.0
Quinolone	85.7	138.5	144.7	152.4	161.5	156.4	168.7	166.2	167.4	277.1	274.6	273.9	272.8	200.1
Rifamycin	0.0	0.0	0.0	0.0	0.0	0.1	0.4	1.2	2.4	4.6	6.7	7.2	8.7	8.8
Sulphonamide	10.0	15.0	14.6	15.1	14.6	15.6	15.3	18.3	18.8	26.9	26.4	27.3	30.3	28.3
Tetracycline	9.2	15.5	16.2	17.1	18.8	21.0	23.9	29.6	50.5	82.0	111.0	141.3	174.0	193.4
Overall use	1,206.3	1,969.4	2,058.9	2,147.5	2,316.5	2,251.0	2,465.2	2,527.0	2,642.4	4,492.4	4,651.0	4,637.6	4,746.9	3,967.6

*DHFRi, dihydrofolate reductase inhibitor.

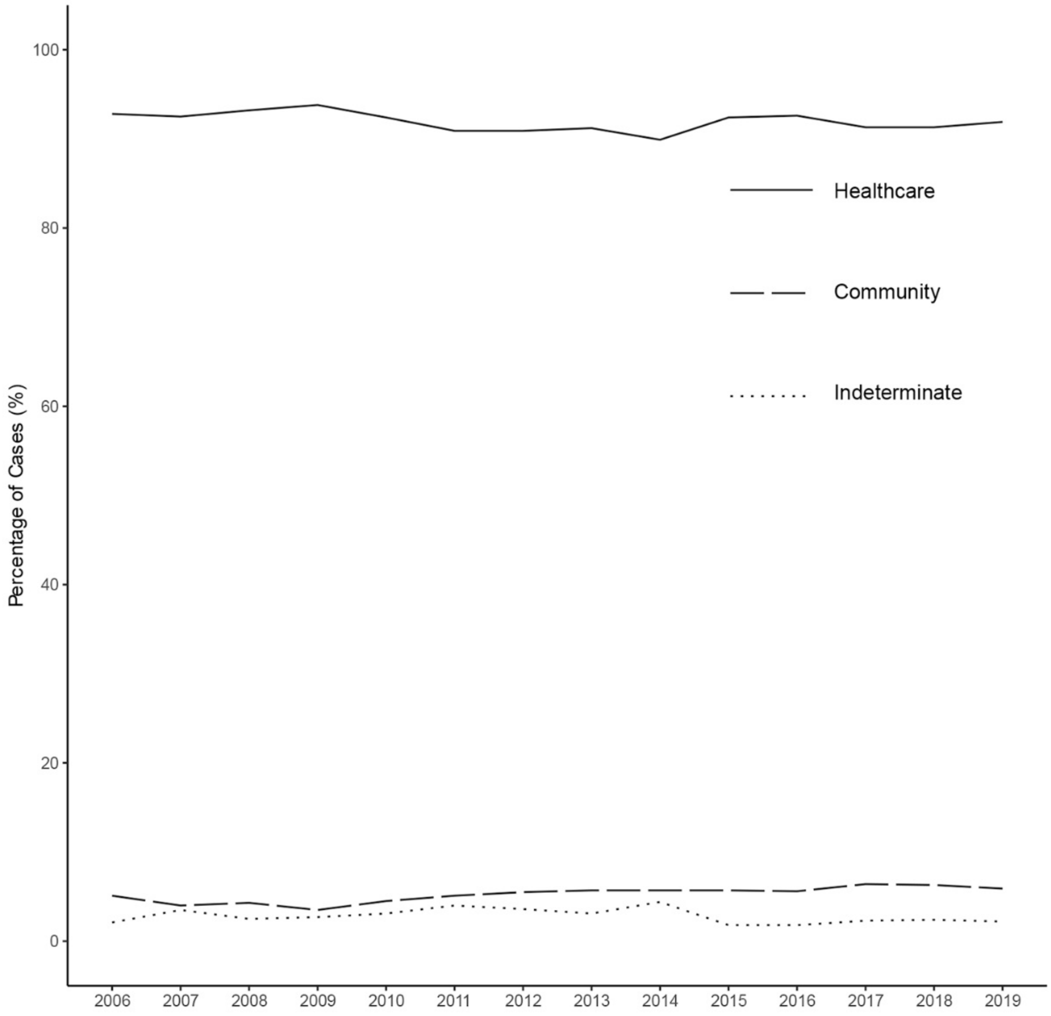

Appendix Figure. Percentage of *Clostridioides difficile* infections cases stratified by epidemiologic class in Hong Kong, China, 2006–2019.
